# Associations between Health Education and Mental Health, Burnout, and Work Engagement by Application of Audiovisual Stimulation

**DOI:** 10.3390/ijerph19159370

**Published:** 2022-07-30

**Authors:** Argang Ghadiri, David-Lennart Sturz, Hadjar Mohajerzad

**Affiliations:** 1 Department of Management Science, Bonn-Rhein-Sieg University of Applied Sciences, Grantham-Allee 20, 53757 Sankt Augustin, Germany; david-lennart.sturz@h-brs.de; 2 German Institute for Adult Education, Leibniz Centre for Lifelong Learning, Heinemannstr. 12-14, 53175 Bonn, Germany; mohajerzad@die-bonn.de

**Keywords:** health education, workplace health promotion, audiovisual stimulation, relaxation, mental health, well-being, work engagement, burnout

## Abstract

Due to the COVID-19 pandemic, health education programs and workplace health promotion (WHP) could only be offered under difficult conditions, if at all. In Germany for example, mandatory lockdowns, working from home, and physical distancing have led to a sharp decline in expenditure on prevention and health promotion from 2019 to 2020. At the same time, the pandemic has negatively affected many people’s mental health. Therefore, our goal was to examine audiovisual stimulation as a possible measure in the context of WHP, because its usage is contact-free, time flexible, and offers, additionally, voice-guided health education programs. In an online survey following a cross-sectional single case study design with 393 study participants, we examined the associations between audiovisual stimulation and mental health, work engagement, and burnout. Using multiple regression analyses, we could identify positive associations between audiovisual stimulation and mental health, burnout, and work engagement. However, longitudinal data are needed to further investigate causal mechanisms between mental health and the use of audiovisual stimulation. Nevertheless, especially with regard to the pandemic, audiovisual stimulation may represent a promising measure for improving mental health at the workplace.

## 1. Introduction

### 1.1. Background

Health education as “any planned combination of learning experiences designed to predispose, enable, and reinforce voluntary behavior conducive to health in individuals, groups or communities” [1] plays an important role to alter healthy behavior. It includes certain aspects with an educational character, such as target-oriented communication activities [2] or learning experiences and the acquisition of information and skills [3,4]. In the workplace setting, employees’ health behavior is addressed by specific measures which can be compromised as workplace health promotion (WHP) (cf. [5] for theoretical reflection). Health education and health promotion therefore go hand-in-hand, since both aim to improve health and well-being of individuals [3,6,7,8].

However, during the COVID-19 pandemic, companies had to face different challenges such as physical distancing, mandatory lockdowns, and isolation periods [9]—circumstances that impede offering adequate WHP. In Germany for example, the statutory health insurance expense for prevention and health promotion fell from 630.8 million Euro (2019) to 414 million Euro (2020) [10], in times where stress, depression, panic, anxiety, or sleep disorders dramatically impacted the mental health status and well-being of individuals [9,11,12,13,14,15].

In the workplace setting, traditional prevention courses are offered by a certified trainer, face-to-face and usually for larger groups of employees, on a weekly basis over a period of 8 to 12 weeks. Those courses cover the main areas of exercise, nutrition, stress management, and substance abuse and aim to motivate and enable participants to acquire new information and skills for their health [16]. Besides these traditional measures, digital prevention courses have gained more attention, which mainly use online video, webinars, or other (instructed) learning forms [17,18].

In this context, audiovisual stimulation (so-called brainwave synchronizer or mind machines) seems to be a promising measure to improve health status and well-being [19]. In its original form, audiovisual stimulation systems influence brain states by external sound and light frequencies provided via headphones and glasses with light-emitting diodes. Those stimuli in turn are adapted by the brain (so-called brain entrainment) and thus can stimulate brain waves into desired stimulation frequencies which induce relaxation and consciousness [20,21]. Fundamental research into audiovisual stimulation has already shown positive effects on anxiety [22,23,24], depression [25,26], headache [27,28,29,30], and sleep disorders [31,32,33].

Especially considering the health protective measures during the pandemic, audiovisual stimulation systems can be used without a trainer and thus can be practiced alone without infection risk. Moreover, modern audiovisual relaxation systems contain a variety of voice-guided health promotion programs and health education courses in combination with the audiovisual stimulation. Those programs are created as instructed courses with sequential units and trainings, such as mindfulness training, autogenic training, substance abuse treatment, etc. [34].

### 1.2. State of Research

Research on audiovisual stimulation in the context of WHP predominantly has been undertaken in the setting of rest breaks. For example, Wollseiffen and colleagues [35] analyzed the effect of audiovisual stimulation on cognitive performance and well-being among other rest-break interventions but could not conclude significant effects among the intervention groups with 10 participants each [35]. In another study with 12 participants, Scholz and colleagues [36] examined the effects of different rest-break interventions in a repeated measurement design, including audiovisual stimulation. Results indicated positive tendencies in general, but significant results regarding audiovisual stimulation could not be obtained [36]. In another study, Singh and colleagues [37] used a laboratory pre-test-post-test design to compare rest-break interventions. Results showed significant improvements in performance and subjective well-being (stress, recovery etc.) after using audiovisual stimulation compared to the intervention condition of napping [37]. Nonetheless, existing studies on audiovisual stimulation in the field of WHP show different weaknesses due to the experimental design of the studies. First, the studies only have small sample sizes of under 20 participants [35,36,37]. Second, research has been undertaken in a laboratory setting, which might have influenced detachment from work during the rest breaks and thus the final results [35,36,37]. Third, only short-term effects after one single usage of the intervention have been examined [35,36,37], although research on audiovisual stimulation suggests long-term usage for sustainable effects [21].

### 1.3. Objectives of the Study

Regarding the pandemic, employees have to face new circumstances with their work, such as physical distancing, mandatory lockdowns, isolation periods, etc. [8], which cause high levels of stress and influence mental health. According to the Job Demands–Resources (JD–R) model [38] each work has certain job-stressors, which can be divided into job demands and job resources. Job demands describe work-associated stressors which cost (physical and psychological) energy, whereas job resources help to balance and counteract stressors. If high job demands cannot be buffered by sufficient job resources in the short-term, this can impact mental health and well-being negatively and lead to burnout in the long-term; on the contrary, high job resources have a motivating effect leading to work engagement [39,40,41]. Based on this model, we consider audiovisual stimulation as a potential job resource to prevent burnout and to increase work engagement because it can positively impact mental health and performance [23,25,26,37].

Therefore, we want to answer the following research questions exploratively by conducting a quantitative single-case study: how is usage of audiovisual stimulation related to (1) mental health and well-being, (2) burnout, and (3) work engagement?

## 2. Materials and Methods

In this section, we describe the sample of the study, as well as the data collection procedure, the self-assessment instrument used, and the statistical analyses we conducted to answer our research questions.

### 2.1. Data Collection and Sample

Data were collected between November and December 2021 through an online survey. This survey followed the design of a cross-sectional single-case study. The used dataset was provided by an organization distributing audiovisual stimulation systems in Germany. The respective email address holders have explicitly consented to storage and contact by the organization. The personal usage of the audiovisual system was not influenced by our study design; we solely asked for retrospective data in our survey. In October 2021, via personal e-mail invitation, 696 employees using any kind of audiovisual stimulation program (prerequisite to be included in the study) received a standardized questionnaire and an invitation letter containing all relevant information about the study. The potential respondents were informed that participation in the study was voluntary, and that all data collected would be anonymized, analyzed, and published. On 31 December, a total of 468 participants had started answering the survey, which resulted in a response quote of 67.24%: a good response rate for studies in the field of business studies [42,43,44,45]. Of these participants, 75 participants were eliminated due to dropping out too early after validating that their responses did not systematically differ from the complete observations, leading to a completion rate of 56.30%.

In absolute numbers, 393 participants (range age = 22 to 88, mean = 53.56) completed the questionnaire, with the largest age groups being 41 to 50 years (*n* = 94, 23.70%), 51 to 60 years (*n* = 170, 42.90%), and 61 to 70 years (*n* = 73, 18.40%). The gender was 44.4% male, 54.5% female; 0 participants reported to be “diverse”, and 1% did not indicate a gender. The majority of respondents were employed (*n* = 199, 50.20%) or self-employed (*n* = 130, 32.80%); only three people worked as freelancers (0.80%), and the remaining participants gave no information (*n* = 77, 19.4%). In total, people working in 17 different sectors participated, whereby the sector classification was based on the classification of economic sectors [46]. The most strongly represented sector was health and social services (*n* = 92, 23.2%). This was followed by the manufacturing industry (*n* = 39, 9.8%) and the public sector (*n* = 26, 6.6%), but 21% (*n* = 83), on the other hand, gave no information regarding their industry affiliation. Of the 293 participants, 278 (70.2%) reported that their companies had workplace health management or other health promotion activities at their workplace. In addition, it was asked whether the employees have been working in their current company for at least one year. The survey also asked about the working time models, the daily working time including overtime, management responsibility, as well as the number of people living in the household and, if applicable, children living in the household (see Table 1).

### 2.2. Instruments

The online survey covered seven main areas, the composition of which is briefly described in this section.

#### 2.2.1. Short-Form-36 Health Survey

The SF-36 (Short-Form-36 Health Survey) is a validated and internationally frequently used multidimensional instrument for the assessment of health-related quality of life. The validated German version of the SF-36 was used for this questionnaire [47]. The questionnaire determines the health-related quality of life through eight scales, which represent eight dimensions of health. The measure items are distributed as follows: physical function (10 items), role physical (4 items), bodily pain (2 items), general health (5 items), vitality (4 items), social function (2 items), role emotion (3 items), and mental health (5 items) (cf. Table 2). The weighted scores of the individual scales correspond to a high score which defines a more favorable state of health. These can be on a scale between 0 and 100 points and at the same time indicate the percentage of the total points achieved. The answers to these 36 statements are given in the form of a five-point Likert scale, ranging from “definitely true” (1) to “definitely false” (5). In a second step, the accumulated points are weighted so that 1 results in a value of 0 and 5 in a value of 100 [48]. These eight different dimensions, in turn, can be interpreted as physical and psychological components through a weighted summation [49,50].

#### 2.2.2. Oldenburg Burnout Inventory

The Oldenburg Burnout Inventory (OLBI) is a validated instrument, especially in a research context, for measuring burnout, independent of the occupational context [51,52,53]. The OLBI measures the main dimensions of burnout, exhaustion, and disengagement from work as separate but interrelated factors [39]. Both subscales are determined by eight questions each, which in turn consist of four positively and four negatively formulated statements. Scores per item range from “strongly agree” (1) to “strongly disagree” (4) on a four-point Likert scale [54].

#### 2.2.3. Utrecht Work Engagement Scale

The Utrecht Work Engagement Scale (UWES) is a validated and frequently used instrument to measure work engagement [55], which is considered to be the opposite of burnout [56]. Work engagement itself is determined by the means of 9 items, namely vigor (3 items), dedication (3 items), and absorption (3 items), the latter representing being completely absorbed in one’s work [57]. The following statements, among others, were included: “I am bursting with energy when I work”, “I feel strong and energetic when I work”, “I am enthusiastic about my work”, “My work inspires me”, “When I get up in the morning, I feel like working”, “I feel happy when I work intensively”, “I am proud of the work I do”, “I am absorbed in my work”, “I get carried away when I work”. The scores per item range from “never” (0) to “always/every day” (6) on a six-point Likert scale [51].

#### 2.2.4. Work-Related Items

The current employment relationship, as well as the work environment, were asked on the base of five items. First, the working time model used by the participants was queried, by giving different answering options. Whether participants were already employed by their current employer before the COVID-19 pandemic was recorded by means of a nominal query (company affiliation). Using a metric scale, the weekly working time was asked once with (compatible workload) and once without overtime (workload with overtime). Management responsibility was determined with the question “Do your current tasks include managing employees or managing a team, a division or part of a company?”. The scores for the individual items were mainly determined nominally (0 = Yes, 1 = No). Only in the case of working time, these items were determined metrically.

#### 2.2.5. Workplace Health Promotion

Whether a WHP exists in the company was determined with the help of one item. It was queried with the question, “Does your company offer workplace health promotion?” The participants had to give the following answer options: “Yes” (0), “No” (2), “I’m not sure” (3).

#### 2.2.6. Average Use Audiovisual Stimulation

The frequency of use of audiovisual stimulation in the context of work was determined by asking the respondents to indicate how often they used audiovisual stimulation on average in a month. The response scale was metric.

#### 2.2.7. Personal Information

Five different items were identified as demographic data in a more restrictive sense. Gender was determined by a selection question with the options “male” (0); “female” (1); and “diverse” (2). The age was determined by entering the date of birth. The number of persons living in the household was asked by entering a metric number. If more than one person lived in the household, the number of children living in the household was also determined by asking for the exact number.

### 2.3. Data Analysis

Overall, data analysis included preliminary confirmatory factor analysis (CFA) to assess the validity of the scale for the dimensions, general health, burnout, and work engagement on our sample. Since we are measuring latent constructs, we used factor scores from the CFA results. Finally, OLS regression analyses were conducted to assess the association between audiovisual stimulation use and health. Additional details on each step of the analysis are provided in the following subsections.

#### 2.3.1. Preliminary Analysis: The Factorial Validity of the Dimensions

Confirmatory factor analyses (CFA) were conducted to validate the structure of the general health, burnout, and work engagement scales. CFA is a statistical procedure to assess construct and factorial validity by testing whether the data fit the hypothesized measurement model.

To estimate general health, we estimated 35 items in eight dimensions, differentiated into physical and mental health [58]. Since the general health scale is hierarchically structured and alpha coefficients can mis-estimate the reliability of the overall scale [59], we assessed factorial validity using a second-order CFA model. The second-order CFA model assumes second-order factors (i.e., physical and mental health) reflecting the overarching construct of physical and emotional health, with direct causal pathways to the first-order factors (i.e., the 35 subscales of the SF-36 Short), measured directly by the 35 items. In this model, the covariance between the first-order latent variables was described by the overall second-order latent construct called “physical health” and “mental health”. Second, we estimated the fit of the 16 items in the two burnout dimensions. The first CFA model was run with the two first-order correlated latent variables (i.e., the 16 burnout subscales). For the work engagement dimension, we estimated eight items in the three dimensions (vigor, dedication, and absorption). For this, we ran the second CFA model with the three first-order correlated latent variables. According to the modification indices, residual correlations were added to improve the model fit of the first- and second-order CFA models. Modifications were made only where appropriate and theoretically well-justified. The fit of the models was assessed with several fit indices, as the chi-square assesses the absolute fit of the model, but can be affected by sample size, correlations, and model-independent variance. Therefore, we also considered the comparative fit index (CFI; acceptable fit ≥0.90; close fit ≥0.95; cf. [60]), the standardized root mean square residual (SRMR; good fit ≤0.08; cf. [61]), and the root mean square error of approximation (RMSEA; acceptable fit ≤0.10 for small samples, cf. [62]). All analyses were conducted with Stata, version 15.

#### 2.3.2. Statistical Analyses

To assess the associations between audiovisual stimulation use and each of the general health, burnout, and work engagement dimensions, multiple regression analyses (OLS) were conducted separately for the 13 subscales. *F*-values (and the respective *p*-values) and adjusted R^2^ values for the regression models were determined for all regression models, while standardized regression coefficients (*β*-values) and their respective *p*-values were determined for the statistically significant predictors. Following Cohen [63], we interpreted the standardized regression coefficients (*β*-values) of 0.10 to 0.29 as indicating a small effect, 0.30 to 0.49 as indicating a moderate effect, and 0.50 as indicating a large effect.

### 2.4. Descriptive Statistics

The general descriptive statistics of all variables are presented in Table 1. Descriptive statistics were generated for frequency of participants’ gender, working time model, company affiliation, managerial responsibility, and WHP category. For the metric variables, means and standard deviations were calculated for the values of the average usage and working time variables as presented in Table 1.

For working time model (*n* = 319), 96.6% (*n* = 308) stated that they have a permanent employment contract, while 3.4% (*n* = 11) had a fixed-term contract. Regarding their company affiliation, 96.2% (*n* = 305) stated that they had already worked for their previous employer before 2020, and thus before the COVID-19 pandemic; 54.5% (*n* = 176) stated that they currently have management responsibility and are therefore the personnel manager of at least one person (*n* = 323); 45.5% (*n* = 147), on the other hand, stated that they do not hold a management position. Regarding WHP (*n* = 327), 85.02% (*n* = 278) said that their company offers one and 14.98% (*n* = 49) knew that their company did not have one. Only 392 indicated gender, of which 44.9% were male and 55.1% were female. 

## 3. Results

After providing some information on the factorial validity of the instrument, we address the three research questions by first presenting the descriptive statistics, then providing the evidence of the association between audiovisual stimulation and general health, burnout, and work engagement.

### 3.1. Results of the Preliminary Analysis: Factorial Validity of the General Health Scale

For the second-order CFA model, we chose the robust maximum likelihood estimation method because the assumptions of normality distribution of the data were violated here as well. The fit indices of the third CFA model were not satisfactory, so we added seven residual correlations to improve model fit by referring to the modification indices when they matched the hypothesized general health model. Only inter-item correlations within the same domains were added. Second-order CFA fitting for 35 items of the general health scale showed a good fit: χ^2^ (540) = 1325.183, RMSEA = 0.061, 90% CI [0.057, 0.065], CFI = 0.905, SRMR = 0.065. Standardized factor loadings ranged from 0.44 to 0.89. Both factor loadings (physical and emotional health) were high (from 0.77 to 0.99 mental health and 0.71 to 0.75 physical health). All factor loadings are shown in Table 2.

### 3.2. Results of the Preliminary Analysis: Factorial Validity of the Burnout Scale

**Figure 1:**
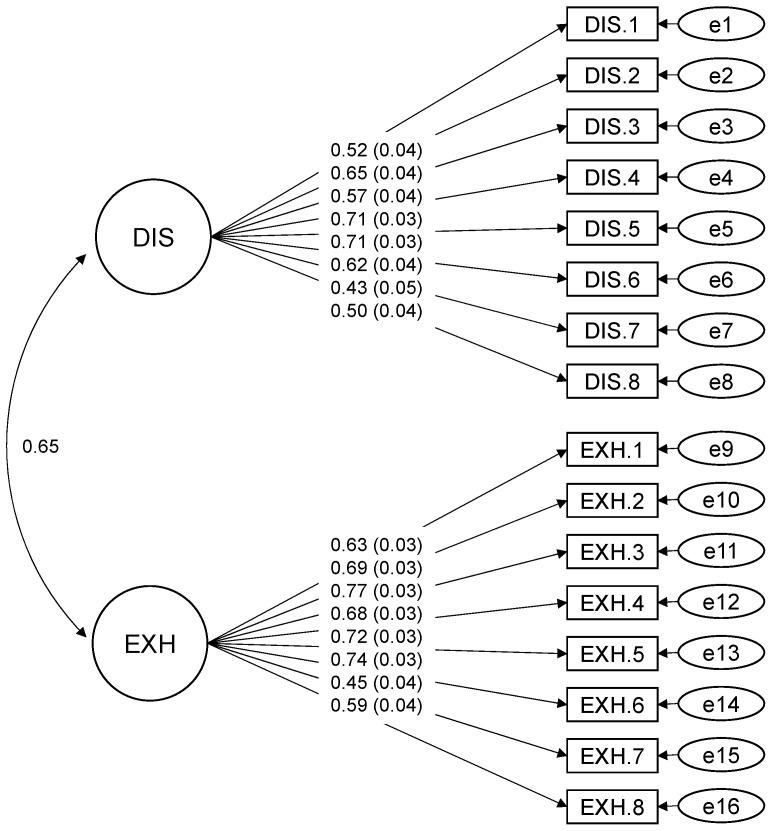
CFA Model of the Burnout Measurement. DIS: disengagement from work, EXH: exhaustion, *n* = 396. Residual correlations between the following pairs of items were added to the model: item DIS.1 with item DIS.3 (=0.353, SE = 0.050, *p* < 0.001); item DIS.2 with item DIS.6 (=0.224, SE = 0.056, *p* < 0.001); item DIS.1 with item DIS.8 (=0.165, SE = 0.04, *p* < 0.001); item DIS.7 with item DIS.8 (=0.279, SE = 0.049, *p* < 0.001); item EXH.2 with item EXH.8 (=0.236, SE = 0.057, *p* < 0.001); item DIS.6 with item DIS.8 (=0.324, SE = 0.057, *p* < 0.001).

We chose the robust maximum likelihood estimation method because the assumptions of normality distribution of the data were violated. The fit indices of the first CFA model were not satisfactory, so we added six residual correlations to improve model fit by referring to the modification indices when they matched the hypothesized burnout model. Only inter-item correlations within the same burnout range were added. As a result, the first-order CFA for the 16 items of the burnout scale showed a good fit: χ^2^ (98) = 343.267, RMSEA = 0.080, 90% confidence interval (CI) [0.071, 0.089], CFI = 0.897, SRMR = 0.079. Although the chi-square test was significant (*p* < 0.001), we did not discard the model because the significance of the test is affected by sample size, while the other fit indices were partially satisfactory. The results confirmed the two-factor structure. The standardized factor loadings ranged from 0.43 to 0.77. All factor loadings are shown in Figure 1.

### 3.3. Results of the Preliminary Analysis: Factorial Validity of the Work Engagement Scale

We also chose the robust maximum likelihood estimation method in this model because the assumptions of normality distribution of the data were violated.

**Figure 2:**
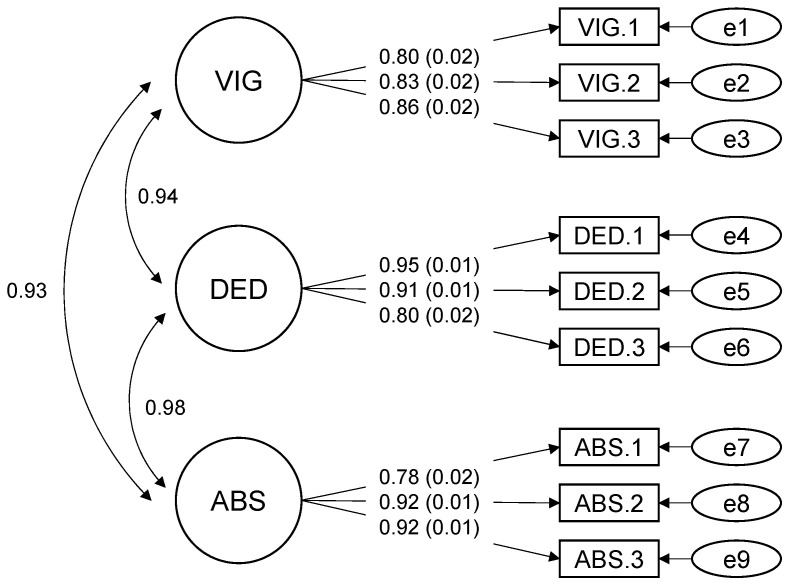
CFA of the Work Engagement Measurement. VIG: vigor, DED: dedication, ABS: absorption, *n* = 395. Residual correlations between the following pair of items were added to the model: item VIG.1 with item VIG.2 (=0.359, SE = 0.051, *p* < 0.001).

We added a residual correlation to improve the model fit by referring to the modification indices. As a result, the CFA for the nine items of the work engagement scale showed a good fit: χ^2^ (22) = 114.245, RMSEA = 0.103, 90% confidence interval (CI) [0.085, 0.122], CFI = 0.975, SRMR = 0.027. Since we have a small sample *n* < 320, the model is acceptable with an RMSEA at 0.10 [62], therefore the fit indices were satisfactory. The results confirmed the three-factor structure. The standardized factor loadings ranged from 0.78 to 0.95. All factor loadings are shown in Figure 2.

### 3.4. Associations between Use of Audiovisual Stimulation and Health

This section provides the answer to the research question. Multiple linear regression analyses were conducted to examine the associations between audiovisual stimulation use and health aspects related to work.

#### 3.4.1. General Health

In order to examine the relationship between the use of audiovisual stimulation and the dimension of general health in the distinction between physical and mental health, eight multiple linear regressions were performed with all the previous independent variables and use of audiovisual stimulation. From Table 3 it can be seen that the association between use of audiovisual stimulation and the factors “physical function”, “bodily pain”, and “social function” is not significant. The factors “physical function”, “bodily pain” and “role physical” measure physical health (PCS) [58], furthermore the association between use of audiovisual stimulation and “role physical” is significant. Therefore, we conclude that no association exists between the use of audiovisual stimulation and physical health. However, for the use of audiovisual stimulation we found statistically significant regression model for the factors of mental health (MCS), such as vitality, role emotion, mental health, and general health; however, all eight subscales have a small amount of explained variance (see Table 3 for details).

#### 3.4.2. Burnout

To investigate the relationship between use of audiovisual stimulation and burnout, multiple linear regressions were run with all the previous independent variables to assess the relationship and predictive power of the “distance from work” factor and the “exhaustion” factor, taking into account the variance of both factors. Table 4 shows the results. For the dependent variable “distance from work”, we found a statistically significant regression model with 14.8% variance explanation (*F* (11, 266), *p* < 0.001). Two statistically significant predictors were identified: respondents’ age (*β* = −0.23, *p* = 0.001) had a small negative effect on the dependent variable (i.e., the older the respondents were, the less the disengagement). The use of audiovisual stimulation also had a small negative effect (*β* = −0.18, *p* = 0.01) on the dependent variable (i.e., the more frequent the use of audiovisual stimulation, the less the disengagement from work).

Regarding the use of audiovisual stimulation for “exhaustion”, a statistically significant regression model with a small amount of explained variance emerged: *F* (11, 266) = 1.66, *p* < 0.001; corrected R^2^ = 0.26. Only the use of audiovisual stimulation was a significant predictor: it had a small negative effect (*β* = −0.19, *p* < 0.01) on the dependent variable (i.e., the more frequent the use of audiovisual stimulation, the less exhausted the respondents felt).

#### 3.4.3. Work Engagement

To examine the dimension of work engagement, we conducted three multiple regression analyses (Table 5). For the dependent variable “vigor”, we found a statistically significant regression model with a 15.06% variance explanation (*F* (11, 266), *p* < 0.001). Five statistically significant predictors were found: working time model (*β* = 0.22, *p* = 0.001), company affiliation (*β* = 0.12, *p* = 0.05), managerial responsibility (*β* = −0.15, *p* = 0.001), age (*β* = 0.18, *p* = 0.001) and the use of audiovisual stimulation (*β* = 0.23, *p* = 0.001, i.e., the more frequent the use, the higher the agreement with vigor).

Regarding the factor “dedication”, a statistically significant regression model was found with a 12.49 percent variance explanation (*F* (11, 266), *p* < 0.001). Three statistically significant predictors were found: use of audiovisual stimulation had a small positive effect (*β* = 0.17, *p* < 0.01) on the dependent variable, working time model had also a small positive effect (*β* = 0.13, *p* < 0.05) and managerial responsibility had a small negative effect (*β* = −0.15, *p* < 0.05).

For the absorption factor, we also found a statistically significant regression model with a 12.84 percent variance explanation (*F* (11, 2660, *p* < 0.001). Four statistically significant predictors were found: managerial responsibility (*β* = −0.17, *p* = 0.01), age (*β* = 0.13, *p* = 0.05), gender (*β* = 0.14, *p* = 0.05), age and the use of audiovisual stimulation (*β* = 0.21, *p* = 0.001, i.e., the more frequent the use, the higher the agreement with being absorbed).

## 4. Discussion

In this study, we aimed to examine the associations of audiovisual stimulation as a measure for health education and WHP. Since previous studies on audiovisual stimulation in the field of WHP had several weaknesses [35,36,37], we conducted a quantitative survey with field data due to several reasons. First, we wanted to obtain data about possible long-term associations of usage in contrast to previous studies which examined solely single-usage of audiovisual stimulation [35,36,37]. Second, previous studies only had sample sizes under 20 participants [35,36,37]. Third, research has been undertaken in laboratory settings and also included students as participants [37]. Nevertheless, audiovisual stimulation showed tendencies to improve productivity and health-related variables [36,37]. Therefore, the focus was to analyze the associations between the usage of audiovisual stimulation systems and health—especially with regard to mental health. Therefore, we also measured burnout on one hand, and subsequent work engagement on the other.

Due to the fact that audiovisual stimulation can be used as a measure to induce relaxation [20,21,22,23,24,25,26,27,28,29,30,31,32], we hypothesized that an association exists between usage and mental states, respectively health. As the results show, associations between audiovisual stimulation and the mental health of employees are significant. Using the SF-36 we found significant effects on the scales of vitality, role emotion, and mental health. Moreover, the audiovisual stimulation system in our study contained health education programs covering topics for healthy lifestyle (e.g., sleep, nutrition, exercise), meditation, and other voice-guided programs to promote health along with the stimulation [34]. This might explain why the association between the usage of the audiovisual relaxation system and mental health is significant and also the scale of general health. However, in line with the literature [18,19], we could not identify significant associations between audiovisual stimulation and the other measures of the SF-36 regarding physical health, namely role physical function, role physical, and bodily pain.

Regarding burnout, we found a statistically significant regression model for audiovisual stimulation on the dimensions “distance from work” and “exhaustion” measured with the OLBI [39]. Results of exhaustion show that the more frequent the respondents use audiovisual stimulation the less exhausted they feel. The same applies to distance from work, the more frequent audiovisual stimulation is used, and the less distant the respondents are from their work. Previous research into audiovisual stimulation could not identify significant associations between audiovisual stimulation and burnout, although positive short-term associations effects on anxiety could be obtained in an experimental study with 42 test persons [23]. In contrast to that, our survey followed a quantitative approach (assuming the positive associations) which enabled us to access a larger database from the field.

Along with burnout, we also examined the association between work engagement and audiovisual stimulation by using the scales of UWES [55]. In doing so, we found statistically significant regression models for “vigor”, “dedication”, and “absorption” associated with the use of audiovisual stimulation, indicating that its use has led to higher work engagement.

Our results substantiate that digitally provided health interventions can be implemented successfully in WHP through technological devices [64,65]. In this context, recent research has shown the importance and effectiveness of internet-based and app-based mobile health interventions to mitigate negative impacts of the COVID-19 pandemic and mental health issues [66,67]. Especially in the healthcare sector, digitalization of WHP seems to be a promising approach since several studies showed the successful integration of web-based interventions [68,69,70]. However, the impact of digital health interventions depends on how the tasks can be implemented in the daily workflow [65] and aspects such as perceived usefulness or enjoyment [64]. Regarding our investigated intervention, audiovisual stimulation can be implemented in the daily workflow like for example within rest breaks [35,36,37] and represents an easy-to-use solution. Sessions on audiovisual systems only require the selection of a certain program using a control panel instead of using an app or mobile device which could induce a certain level of complexity for the user. This could also explain the positive associations between the audiovisual stimulation and our investigated measures.

### Limitations and Future Study Directions

Regarding the main limitations of the study, given the cross-sectional design, we cannot claim causality, only associations. In addition, although an examination of the frequency of audiovisual stimulation use during work was examined, the data do not allow for an examination of the intensity of audiovisual stimulation use. Longitudinal data are needed to further investigate causal mechanisms between mental health and the use of audiovisual stimulation and how these could be used in the context of WHP and health education.

In addition to that, future research should also differentiate between audiovisual stimulation as a WHP measure and as a health education program on its own in order to distinguish possible differences and effects. Regarding physical health, it would be interesting to evaluate effects of voice-guided health education programs in the field of exercise to obtain information on how such programs can influence healthy behavior with respect to exercise.

Recent research has also pointed out that the integration of social components and strategies can be beneficial the acceptance and effectiveness of digital measures in WHP, including for example an exchange with professionals [66]. Blended approaches might therefore be of great interest for future research, since solely digitally distributed measures can lead to isolation and impact intended effects of the measure negatively [67].

It is quite likely that our sample represents a “positive selection” in terms of attitudes towards audiovisual stimulation; however, the frequency of audiovisual stimulation use on health will be investigated. Even if positive attitudes toward audiovisual stimulation would correlate with usage, this does not mean that it would have an impact on the association between the use of audiovisual stimulation and mental health. This would certainly be worth investigating in further research as a mediator effect. Since this study examined the use of audiovisual stimulation it seems plausible in further studies to develop a usage scale with more usage items, which could be a reason for the partial low variance resolution.

Moreover, due to the lack of previous studies we analyzed associations between audiovisual stimulation and health, work engagement, and burnout by using the correspondent subscales of the measures. Along with our explorative study design, we thereby aimed to reveal as many relations as possible. Regardless, future research should also focus on analyzing the whole scales for better comparability of study results.

In general, this study counters the observation that too little importance is attached to audiovisual stimulation in the context of WHP [35,36,37]. We believe that an expansion of research on the use of audiovisual stimulation and its effectiveness is needed, especially in times when working from home and physical distancing dominate the workplace.

## 5. Conclusions

To our knowledge, our study is the very first to examine the associations of audiovisual stimulation as a WHP measure in a quantitative survey. Previous research implementing audiovisual stimulation in WHP has only analyzed short-term effects on health and productivity in the context of rest breaks [35,37], neglecting possible effects in the long-term. Our study could show significant associations between the usage of audiovisual stimulation and the improvement of (mental) health, work engagement and consequently reducing the risk of burnout, albeit multi-causal effects may have impacted the results on health and work engagement.

However, as working from home, social distancing [71], and economic uncertainty [72] impact the mental health of employees dramatically during the pandemic, new measures for WHP and health education are necessary and strongly needed. Digital solutions have already shown their usefulness during the pandemic [73]; however, more research on digital prevention programs and their acceptance is necessary to identify drivers of user acceptance [17]. In this context, audiovisual stimulation seems to be a promising measure combining digital voice-guided health courses with the effects of audiovisual stimulation as a relaxation promoting measure.

## Figures and Tables

**Table 1:** Descriptive Statistics of Study Variables.

Item-No.	Text	*n* (Valid)	Min	Max	Mean	SD
**Average Audiovisual Stimulation**						
16.1	Average Usage	396	0	35	4.91	7.63
**Work Related Items**						
17.1	Working Time Model ^a^	319	0	1	-	-
17.2	Employment ^b^	396	0	3	-	-
17.3	Company Affiliation (>1) ^c^	305	0	1		
17.4	Compatible Workload	396	0	70	31.17	15.29
17.5	Workload with Overtime	396	0	80	35.71	19.26
17.6	Managerial Responsibility ^d^	323	0	1	-	-
**Workplace Health Promotion**						
18.1	Workplace Health Promotion ^e^	327	0	1	-	-
**Personal Information**						
19.1	Age	396	20	88	68.47	10.256
19.2	Gender ^f^	392	0	1	-	-
19.3	People per Household	396	0	31	2.58	1.89
19.4	Children per Household	396	0	6	0.68	1.03

Notes: SD = standard deviation. ^a^: 0 = temporary contract, 1 = permanent contract; ^b^: 0 = freelancer, 1 = worker, 2 = employed, 3 = self-employed; ^c^: 0 = yes, 1 = no; ^d^: 0 = yes, 1 = no; ^e^: 0 = yes, 1 = no; ^f^: 0 = male, 1 = female, 2 = inter, 3 = divers.

**Table 2:** Results from the CFA of the General Health Measurement.

Item-No.	Text	λ	SE	*p*
**Factor One: Physical Function (PF)**				
1.1	PF.1	0.668	0.032	<0.001
1.2	PF.2	0.667	0.031	<0.001
1.3	PF.3	0.657	0.031	<0.001
1.4	PF.4	0.790	0.022	<0.001
1.5	PF.5	0.756	0.026	<0.001
1.6	PF.6	0.647	0.032	<0.001
1.7	PF.7	0.751	0.025	<0.001
1.8	PF.8	0.782	0.022	<0.001
1.9	PF.9	0.709	0.028	<0.001
1.10	PF.10	0.598	0.035	<0.001
**Factor Two: Role Physical (RP)**				
2.1	RP.1	0.805	0.022	<0.001
2.2	RP.2	0.747	0.027	<0.001
2.3	RP.3	0.847	0.020	<0.001
2.4	RP.4	0.790	0.023	<0.001
**Factor Three: Bodily Pain (BP)**				
3.1	BP.1	0.984	0.037	<0.001
3.2	BP.2	0.674	0.038	<0.001
**Factor Four: General Health (GH)**				
4.1	GH.1	0.776	0.026	<0.001
4.2	GH.2	0.559	0.040	<0.001
4.3	GH.3	0.481	0.043	<0.001
4.4	GH.4	0.441	0.045	<0.001
4.5	GH.5	0.832	0.023	<0.001
**Factor Five: Vitality (VT)**				
5.1	VT.1	0.665	0.031	<0.001
5.2	VT.2	0.709	0.028	<0.001
5.3	VT.3	0.796	0.022	<0.001
5.4	VT.4	0.647	0.033	<0.001
**Factor Six: Social Function (SF)**				
6.1	SF.1	0.607	0.038	<0.001
6.2	SF.2	0.880	0.033	<0.001
**Factor Seven: Role Emotional (RE)**				
7.1	RE.1	0.699	0.031	<0.001
7.2	RE.2	0.832	0.023	<0.001
7.3	RE.3	0.818	0.023	<0.001
**Factor Eight: Mental Health (MH)**				
8.1	MH.1	0.605	0.035	<0.001
8.2	MH.2	0.786	0.024	<0.001
8.3	MH.3	0.677	0.030	<0.001
8.4	MH.4	0.745	0.026	<0.001
8.5	MH.5	0.690	0.029	<0.001
**Factor Nine: Physical Component Summary (PCS)**				
PF	Physical Function	0.718	0.038	<0.001
RP	Role Physical	0.749	0.038	<0.001
BP	Bodily Pain	0.745	0.043	<0.001
GH	General Health	0.538	0.069	<0.001
**Factor Ten: Mental Component Summary (MCS)**				
VI	Vitality	0.989	0.026	<0.001
SF	Social Function	0.820	0.038	<0.001
RE	Role Emotional	0.775	0.031	<0.001
MH	Mental Health	0.953	0.026	<0.001
GH	General Health	0.354	0.068	<0.001

Factor loadings (λ) and standard errors (SE) resulting from CFA, *n* = 396. Residual correlations between the following pairs of items were added to the model: item PF.1 with item PF.5 (=0.442, SE = 0.054, *p* < 0.001); item PF.2 with item PF.3 (=0.437, SE = 0.043, *p* < 0.001); item PF.7 with item PF.8 (=0.583, SE = 0.037, *p* < 0.001); item VT.1 with item VT.2 (=0.722, SE = 0.026, *p* < 0.001); item MH.1 with item MH.3 (=0.366, SE = 0.044, *p* < 0.001); item MH.3 with item MH.5 (=0.281, SE = 0.046, *p* < 0.001).

**Table 3:** Multiple Linear Regression on all General Health Subscales.

	Physical Function		Role Physical		Bodily Pain		General Health		Vitality		Social Function		Role Emotion		Mental Health	
	β	SE	β	SE	β	SE	β	SE	β	SE	β	SE	β	SE	Β	SE
Audiovisual Stimulation	0.048	0.135	0.133 *	0.264	0.088	0.256	0.212 ***	0.146	0.216 ***	0.102	0.091	0.237	0.135 *	0.269	0.239 ***	0.137
Working Time Model ^a^	0.163 **	5.448	0.082	10.611	0.065	10.312	0.076	5.882	−0.052	4.114	0.011	9.556	0.285 ***	10.823	0.219 ***	5.512
Company Affiliation (>1) ^b^	0.012	5.610	0.025	10.925	0.036	10.618	−0.068	6.057	−0.055	4.236	−0.068	9.839	0.017	11.144	0.030	5.676
Compatible Workload	−0.046	0.166	0.132	0.323	0.219 *	0.314	0.110	0.179	0.114	0.125	0.052	0.291	0.089	0.326	0.155	0.168
Workload with Overtime	0.137	0.116	−0.113	0.226	−0.116	0.220	−0.094	1.25	−0.164	0.088	−0.123	0.204	−0.043	0.231	−0.167	0.118
Managerial Responsibility ^b^	−0.025	2.228	−0.063	4.339	−117	4.217	−0.110	2.405	−0.109	1.682	−0.102	3.907	−0.036	4.425	−0.043	2.254
Workplace Health Promotion ^b^	0.045	2.844	0.030	5.540	−0.038	4.217	−0.037	3.071	0.019	2.147	0.076	4.999	0.033	5.650	−0.013	2.878
Age	−0.014	0.111	0.090	0.216	−0.056	0.210	−0.068	0.120	0.060	0.084	−0.017	0.194	0.083	0.220	0.036	0.112
Gender ^c^	−0.045	2.147	−0.095	4.181	−0.098	4.063	0.061	2.318	−0.062	1.621	−0.111	3.765	−0.153 *	4.265	−0.151 *	2.172
People per Household	−0.088	0.562	−0.173 *	1.094	−0.017	1.063	−0.012	0.606	−0.007	0.424	−0.066	0.985	−0.070	1.116	−0.005	0.568
Children per Household	0.089	1.165	0.096	2.269	0.073	2.205	0.058	1.258	−0.018	0.880	−0.010	2.043	−0.202	2.312	−0.046	1.179
R^2^ (adj. R^2^)	0.056 (0.018)		0.079 (0.041)		0.082 (0.044)		0.091 (0.054)		0.085 (0.048)		0.047 (0.008)		0.143 (0.108)		0.140 (0.104)	

Notes: * *p* < 0.05; ** *p* < 0.01; *** *p* < 0.001; *n* = 278; *β* = standardized regression coefficient; SE = standard error; ^a^: 0 = temporary contract, 1 = permanent contract; ^b^: 0 = yes, 1 = no; ^c^: 0 = male, 1 = female.

**Table 4:** Multiple Linear Regression on Burnout.

	Distance from Work		Exhaustion	
	β	SE	β	SE
Audiovisual Stimulation	−0.181 **	0.006	−0.187 **	0.006
Working Time Model ^a^	−0.066 **	0.182	−0.010	0.234
Company Affiliation (>1) ^b^	−0.032	0.187	−0.012	0.241
Compatible Workload	−0.094	0.006	−0.139	0.007
Workload with Overtime	−0.055	0.004	0.150	0.005
Managerial Responsibility ^b^	0.082	0.074	0.046	0.096
Workplace Health Promotion ^b^	0.005	0.095	0.064	0.122
Age	−0.231 ***	0.004	−0.087	0.005
Gender ^c^	−0.017	0.072	−0.054	0.092
People per Household	0.077	0.019	0.040	0.024
Children per Household	−0.045	0.039	0.022	0.050
R^2^ (adj. R^2^)	0.141 (0.105)		0.064 (0.026)	

Notes: ** *p* < 0.01; *** *p* < 0.001; *n* = 278; *β* = standardized regression coefficient; *SE* = standard error; ^a^: 0 = temporary contract, 1 = permanent contract; ^b^: 0 = yes, 1 = no; ^c^: 0 = male, 1 = female.

**Table 5:** Multiple Linear Regression on Work Engagement.

	Vigor		Dedication		Absorption	
	β	SE	β	SE	β	SE
Audiovisual Stimulation	0.222 ***	0.010	0.176 **	0.011	0.211 ***	0.011
Working Time Model ^a^	0.217 ***	0.408	0.128 *	0.428	0.103	0.451
Company Affiliation (>1) ^b^	0.115 *	0.416	−0.011	0.440	0.077	0.465
Compatible Workload	0.069	0.012	0.003	0.013	0.037	0.014
Workload with Overtime	−0.013	0.009	0.125	0.009	0.107	0.010
Managerial Responsibility ^b^	−0.148 *	0.165	−0.150 *	0.175	−0.169 **	0.185
Workplace Health Promotion ^b^	−0.094	0.211	0.009	0.223	−0.010	0.236
Age	0.185 ***	0.008	0.186 ***	0.009	0.129 *	0.009
Gender ^c^	0.084	0.159	0.104	0.168	0.144 *	0.178
People per Household	0.029	0.042	0.042	0.044	0.053	0.047
Children per Household	−0.006	0.086	−0.004	0.091	0.013	0.097
R^2^ (adj. R^2^)	0.184 (0.151)		0.160 (0.125)		0.163 (0.128)	

Notes: * *p* < 0.05; ** *p* < 0.01; *** *p* < 0.001; *n* = 278; *β* = standardized regression coefficient; *SE* = standard error; ^a^: 0 = temporary contract, 1 = permanent contract; ^b^: 0 = yes, 1 = no; ^c^: 0 = male, 1 = female.

## Data Availability

The data presented in this study are available on request from D.-L.S.
